# Globalization and social determinants of health: The role of the global marketplace (part 2 of 3)

**DOI:** 10.1186/1744-8603-3-6

**Published:** 2007-06-19

**Authors:** Ronald Labonté, Ted Schrecker

**Affiliations:** 1 Department of Epidemiology and Community Medicine, Faculty of Medicine and Institute of Population Health, University of Ottawa, Canada; 2 Department of Epidemiology and Community Medicine, Faculty of Medicine and Institute of Population Health, University of Ottawa, Canada

## Abstract

Globalization is a key context for the study of social determinants of health (SDH): broadly stated, SDH are the conditions in which people live and work, and that affect their opportunities to lead healthy lives.

In the first article in this three part series, we described the origins of the series in work conducted for the Globalization Knowledge Network of the World Health Organization's Commission on Social Determinants of Health and in the Commission's specific concern with health equity. We identified and defended a definition of globalization that gives primacy to the drivers and effects of transnational economic integration, and addressed a number of important conceptual and methodological issues in studying globalization's effects on SDH and their distribution, emphasizing the need for transdisciplinary approaches that reflect the complexity of the topic.

In this second article, we identify and describe several, often interacting clusters of pathways leading from globalization to changes in SDH that are relevant to health equity. These involve: trade liberalization; the global reorganization of production and labour markets; debt crises and economic restructuring; financial liberalization; urban settings; influences that operate by way of the physical environment; and health systems changed by the global marketplace.

## Background

Globalization is a key context for the study of social determinants of health (SDH): broadly stated, SDH are the conditions in which people live and work, and that affect their opportunities to lead healthy lives. In the first article in this three part series, we described the origins of the series in work conducted for the Globalization Knowledge Network (GKN) of the World Health Organization's Commission on Social Determinants of Health and in the Commission's specific concern with health equity. We identified and defended a definition of globalization that gives primacy to the drivers and effects of transnational economic integration, and addressed a number of important conceptual and methodological issues in studying globalization's effects on SDH and their distribution, emphasizing the need for transdisciplinary approaches that reflect the complexity of the topic.

In this second article, we identify and describe several, often interacting clusters of pathways that lead from globalization to changes in SDH that are relevant to health equity and provide an analytical starting point for more context-specific research. These involve: trade liberalization; the global reorganization of production and labour markets; debt crises and economic restructuring; financial liberalization; urban settings; influences that operate by way of the physical environment; and health systems changed by the global marketplace. These clusters were selected based on the authors' many years of research on how policies adopted by the Group of 8 (G8) countries affect population health outside the industrialized world, and on a review of the relevant literature carried out as preparation for the activities of the GKN.

A theme that emerges consistently across clusters is the "asymmetrical" character of contemporary globalization and its impacts, and the increasingly unequal distribution within and across national borders of gains, losses, and ability to influence globalization's outcomes. The vocabulary is borrowed from Nancy Birdsall, Director of the Center for Global Development, who observed that "globalization, as we know it today, is fundamentally asymmetric. In its benefits and its risks, it works less well for the currently poor countries and for poor households within developing countries" [1]. She was writing mainly about trade policy, where this observation has special force because, under conditions of liberalized trade, labour markets tend to reward those who already possess substantial 'human capital' or the means to acquire it. However, the observation applies as well to financial crises, which tend to exacerbate existing patterns of advantage and disadvantage; to environmental hazards, to which the poor and otherwise marginalized are systematically more vulnerable; and to the more general shift away from political accountability, admittedly often imperfect, and toward economic accountability to the holders of property rights that is a central element of contemporary globalization [2](p. 31–58). This underlying logic of asymmetry links the range of findings reported, in summary form and with illustrative examples, in the sections that follow. It also provides the basis for the generic policy prescriptions that we outline in the third and final article of the series.

### Cluster 1: Trade liberalization, growth and poverty reduction

Perhaps the most familiar element of contemporary globalization is trade liberalization: the lowering of tariffs and other barriers to imports that has been a defining characteristic of the post-World War II economic order. As a consequence, the value of world trade doubled from 24 percent of world gross domestic product (GDP) in 1960 to 48 percent in 2003 [3](accessed March 17, 2007). The argument that globalization is beneficial in terms of population health [4] often starts from an equation of globalization with trade liberalization, and invokes comparative studies on national economies carried out under the auspices of the World Bank [5-7] which concluded that during the 1980s and 1990s, the economies of "globalizers" grew faster than "non-globalizers," thereby expanding the resources at their disposal to provide health services and improve access to other SDH, notably through reduction of extreme poverty.

This conclusion is fragile on several counts. Countries held up as model high-performing globalizers (China, India, Malaysia, Thailand and Viet Nam) actually started out as more closed economies than those non-globalizers whose economies stalled or declined, mostly in Africa and Latin America [7]. The problem is one of definition. Globalizers in these studies are defined as countries that saw their trade/GDP ratio increase since 1977; non-globalizers are simply those that saw their ratio drop. Thus India and China are considered globalizers, even though their trade/GDP ratios at the end of the study period were lower than the average of all countries studied. Conversely, the non-globalizers started out more highly integrated into the world economy. The positive globalization to growth relationship becomes a questionable artefact of the studies' design.

Another critique starts from the premise that economic problems of the non-globalizers are at least partly attributable to global factors outside the control of national economic policy-makers: specifically, a decline in commodity prices that damaged both the export performance and the ability to import of countries that were heavily reliant on commodity exports, but already highly integrated into the global economy on some measures [8-10]. (The decline in commodity prices was partly an effect of other policies that drove countries into intensified export competition with one another in order to pay their debts to external creditors, although this point cannot be explored further here.) Further, excluding India and China from the sample – each of which is arguably a special case, albeit for different reasons – actually changes the conclusion: globalizers grew more slowly than non-globalizers over the period 1980–2000 [10].

We accept as given the proposition that poverty (both absolute and relative) is inimical to health equity and undermines access to SDH. This point is important because, to the extent that globalization is associated with growth, it would appear to be a good thing for population health *if *growth reliably reduces poverty without other offsetting negative consequences. However, similar methodological limitations have been pointed out with respect to this claim [11]. Added concerns exist about the reliability of data on incomes and household assets and the appropriateness of the World Bank's definitions of poverty with reference to poverty lines or thresholds of US $1/day and $2/day [12], especially in large metropolitan areas [13]. Van Doorslaer and colleagues have recently shown, for 11 Asian countries, that surveys on which the World Bank estimates are based understate the extent of poverty as measured by the $1/day poverty line because they are based on surveys of the value of household consumption that include out-of-pocket health care costs. Ironically, then, large numbers of households appear to have escaped poverty because of catastrophic medical expenses [14].

Even if for the sake of argument one takes as given the World Bank measures of poverty, it is not at all clear that globalization leads to substantial poverty reduction. Between 1981 and 2001, the number of people in the world living on $1/day or less fell by 392 million, but the number of people living on $2/day or less rose by 285 million, indicating only that the economically desperate are getting slightly less desperate [11](p. 183). Excluding China, where the accuracy of poverty data has been questioned [15], World Bank data show that the number of global poor actually rose by 30 million at the $1/day level and 567 million at the $2/day level; in sub-Saharan Africa, the number of people living on $1/day or less doubled between 1981 and 2001 (from 164 million to 313 million), and the number living on $2/day or less almost doubled (from 288 million to 516 million). Moreover, half of China's estimated poverty decline occurred from 1981 – 1984, before that country's domestic social policy changes and embrace of the global marketplace, and has been attributed to land reform that "gave farmers considerably greater control over their land and output choices" [11](p. 184),[16].

More detailed attention is not devoted here to debates about trade and growth performance for three reasons.

First, a more fundamental critique of growth as a route to poverty reduction, which stands on its own apart from issues of trade policy, arises from calculations by the New Economics Foundation showing that growth is a very ineffective way of reducing poverty. "Of every $100 of growth in income per person in the world as a whole between 1981 and 2001, just $1.30 contributed to reducing poverty as measured by the $1-a-day line, and a further $2.80 to reducing poverty between $1-a-day and $2-a-day lines"; furthermore, the effectiveness of growth in reducing poverty declined in the 1990s relative to the 1980s [17](p. 16). This is not just an academic point: recent studies of social policy in Latin America [18-20] concluded that even a little redistribution of income through progressive taxation and targeted social programs would go farther in terms of poverty reduction than many years of solid economic growth, because of the extremely unequal distribution of income and wealth in most countries in the region.

Second, trade liberalization is usually just one element of a package of market-oriented economic policy measures that together increase the economic vulnerability of large numbers of people, making its effects difficult to isolate from those of other globalization processes that are occurring simultaneously. These policy measures have often been actively promoted by the industrialized countries and the international institutions they dominate, and the overall implications are explored in greater detail later in this article and in the third and final article in the series.

Third, even the most ardent enthusiasts of trade liberalization concede that there will be direct economic losers: for example, households whose livelihoods in Zambian manufacturing, Ghanaian poultry production, or (in some cases) Mexican corn farming were destroyed by low-cost imports [21-23]. Even when exports are growing rapidly, survival of existing industries may depend on such measures as "downsizing and labour shedding" [24](p. 6). Dismissing those who lose their livelihoods as "inefficient incumbents facing increased foreign competition" [25](p. 96) is an inadequate response to the potential health equity consequences of such transitions. This suggests the need for a generic focus on labour markets (on which trade policy is just one influence among many) and the global reorganization of production as pathways leading from globalization to changes in access to SDH and in health outcomes.

### Cluster 2: Labour markets and global reorganization of production

Along with trade liberalization, and enabled by it, a key element of globalization is the reorganization of production and service provision across multiple national borders by transnational corporations (TNCs) [26-28]. Early research on this topic described a "new international division of labour," in which labour-intensive manufacturing operations were relocated outside the industrialized countries to low-wage jurisdictions, often to Export Processing Zones (EPZs) that offered a variety of special incentives for foreign investors [29]. More recent research on globalization and labour markets emphasizes at least three, related phenomena.

First, a genuinely global labour market is gradually emerging, driven in part by the integration of India, China and the former transition economies into the global marketplace [30-32].

Second, as national jurisdictions compete for foreign direct investment (FDI) and outsourced production, the need to appear 'business-friendly' may limit governments' ability to adopt and implement labour standards, health and safety regulations, and other redistributive social policy measures [33]. Documented examples are difficult to find, which is not surprising since the policy constraint can be expected to operate by way of the mechanism of anticipated reaction. (One exception involves Chinese government proposals to expand the country's very limited labour rights which, as of late 2006, were openly opposed by US manufacturing firms that are major investors in China [34] even though considerable scepticism about the effectiveness of such labour reforms would be in order based on recent Chinese history.) Cerny has captured the potential effects of this dynamic by claiming that globalization will drive convergence of national social and economic policies toward the ideal of what he calls the competition state, focused on "promotion of economic activities, whether at home or abroad, which will make firms and sectors located within the territory of the state competitive in international markets" [35](p. 136).

Third, production is being fragmented and reorganized across multiple national borders in global commodity chains or value chains [36-41], in which each element of production is located where it contributes most to overall returns while reducing financial risks. An important element of this process is 'outsourcing,' in which production is undertaken not by subsidiaries or affiliates of a parent TNC, but rather by notionally independent contract manufacturers and service providers [42] – what might be thought of as the Nike model, after the athletic shoe firm that pioneered it [43,44]. Among the important consequences for research on globalization: even figures such as the estimate, now several years old, that intra-firm trade between various subsidiaries of TNCs, and between subsidiaries and the parent firm, accounts for one-third of the value of global trade [45](p. 153) substantially understate the extent of cross-border integration of production because they do not capture the growing volume of outsourced (sub-contracted) production [42].

The case of Mexico's *maquiladora *export-oriented manufacturing plants and zones is often cited to illustrate some consequences of pursuing integration into global value chains: growing economic and social inequalities among workers [46]; falling wages and deteriorating working conditions for many or most workers [47,48]; eventual loss of some jobs to jurisdictions, notably China, which can offer even lower labour costs [49]; increased workplace hazards and industrial pollution exposure to which is in turn related to labour market position [50-52]. These are not the only effects of economic integration, and research in other countries emphasizes that distribution of gains and loses will depend on the niches that individual workers, firms and national economic policies are able to carve out in global value chains [53,54]. Substantial opportunities for employment and income gains exist, but: "Global value chain pressures are [also] associated with increasing casualization of labour and excessive hours of work" [54](p. 25). Worldwide, the winners will usually be firms and workers with access to the necessary financial resources, skills ('human capital'), and technology. Meanwhile, formerly valuable skills and equipment cannot always quickly, frictionlessly or affordably be replaced by those most relevant to the new global marketplace and some workers, firms, industries and regions will almost inevitably be left behind [24,55].

In terms of effects on income and economic (in)security, one of the most widely noted effects of global integration of production is the sharp decline in the wages of, and demand for, so-called low-skilled workers that has been associated with deindustrialization in the rich countries [56]. International relations scholar Robert Cox has argued without reference to specific country data that globalization divides labour forces into a hierarchical structure of "integrated, precarious, and excluded" workers [57]. This typology is validated by 1997 survey data from Brazil, Chile, Colombia, Costa Rica, El Salvador, Mexico, Panama and Venezuela showing that "the occupational structure has become the foundation for an unyielding and stable polarization of income," with lower income personal service, agricultural, commercial and industrial workers making up 74 percent of the working population; an intermediate stratum of technicians and administrative employees 14 percent, and higher-income professionals, employers and managers just 9 percent [58](p. 61–91). Although connecting this outcome with globalization necessarily involves country-specific investigation, the analysis of these data by the United Nations Economic Commission on Latin America and the Caribbean (ECLAC) links "the need to participate competitively in the world economy" to labour market deregulation, increased flexibility, and the growth of economic insecurity [58](p. 93–102).

Labour markets' tendency to magnify inequality in the context of globalization is not confined to one region. Recently, the World Bank has conceded that despite its optimistic predictions for global growth and the expansion of a global middle class, labour market changes will lead to increased economic inequality in countries accounting for 86 percent of the developing world's population over the period until 2030, with the "unskilled poor" being left farther behind [31](p. 67–100), even before taking into account the shift of income shares from labour to capital that is evident in many national economies. This shift can be substantial. In Mexico the proportion of GDP going to wages fell from 40 percent in 1976 to 18.9 percent in 2000, during a period of rapid integration into the global economy and two major economic crises [59](p. 15–16). Between 1980 and 2006 wages as a share of national income in the G10 countries' GDP fell from almost 63% to just under 59%, while corporate profits in the G7 countries roles from 13 percent to roughly 15.5 percent [32](p. 6–7). Although we have been unable to locate satisfactory data on trends in the distribution of national product between capital and labour worldwide or by region, this pattern is what one might expect given the increasing mobility, and therefore bargaining power, of capital relative to most forms of labour. Workplace health is a related issue, and an extensive review of studies published as of the late 1990s identified a clear preponderance of findings that precarious or contingent work is associated with deteriorations in health and safety protections [60,61] – an especially important observation in view of the worldwide growth in such employment [62].

Like many other aspects of globalization [63-67], its transformation of labour markets affects men and women differently [68]. Although poor working conditions for women are cited as a characteristic of *maquiladora *employment [69-71], observers of export-oriented employment elsewhere in the world argue: "The reality is that, for many women, working in exports is better than the alternatives of working (or being unemployed) in the domestic economy" [67](p. 2); see also [72-74]. A United Nations Research Institute for Social Development (UNRISD) study of export-oriented employment in South Korea, China, Mexico, Mauritius, South Africa, and India found it to be associated with some economic gains for women, in terms both of labour incomes and of work-related entitlement to benefits [68]. However, these gains tend to be disproportionately vulnerable both to economic crises and to systemic, globalization-related pressures for "labour market 'flexibility' and fiscal restraint" [75](p. 25). As suggested earlier, the work itself may have destructive health consequences: women in Bangladesh "do not necessarily expect to work in garment factories for a prolonged period. Indeed, given the toll taken on their health by long working hours, it would not be possible to undertake such work for an extended period of time" [76](p. 151). Thus, whatever economic opportunities globalization has opened up in such cases, they may be available only at the price of exposure to hazardous working conditions.

The UNRISD study is one illustration among many of the need to consider the interplay among multiple elements and consequences of globalization. Its South Korean case study, for example, emphasizes the relations among labour markets, social policy, trade agreement commitments, and responses demanded by the International Monetary Fund to the financial crises of 1997–98 [77]. Mexico actively embraced economic integration well before trade liberalization was entrenched through the North American Free Trade Agreement (NAFTA). It did so partly as a response to the first of a series of financial crises (a temporary default on foreign debt in 1982) the origins of which were themselves global, or at least multinational, but the global diffusion of free-market ideology likewise played a role [78,79]. Drastic currency depreciation associated with Mexico's financial crises, which persisted in spite of policies adopted in response, magnified the direct effects of labour markets on declines in purchasing power and economic polarization within Mexican society [80-83]. This is just one example of how trade liberalization, the new international division of labour and other elements of globalization are bound up with international financial integration and debt crises.

### Cluster 3: Debt crises and marketization under pressure

A long history of debt crises constrains the ability of many developing countries to meet basic needs in the areas of public health, education, water, sanitation and nutrition. Recently, debt service payments have contributed to a larger pattern of financial transfers from the South to the North, most importantly the United States, which contradicts colloquial wisdom about the direction of global financial flows [84]. The etiology of debt crises varies from country to country and over time [85-88], but a stylized list of major causes includes: (a) the oil price shocks of 1973 and 1979–80, which had an especially severe impact on low-income, oil-importing countries; (b) aggressive lending by banks seeking to invest deposits from oil-exporting countries (c) a rapid increase in real interest rates during the early 1980s generated by the monetary policies of the US Federal Reserve, meaning that debtor countries often had to roll over existing debt at much higher interest rates; (d) falling world prices, i.e. deteriorating terms of trade, for the primary commodities that are the key exports of many developing economies; and (e) capital flight, consisting both of outright theft and of the rational, mostly legal shifting of assets abroad by economic elites worried about tax increases and future devaluations.

In the context of globalization and its asymmetries, capital flight assumes special importance. Economic historian Thomas Naylor [87] has commented that: "There would be no 'debt crisis' without large-scale capital flight" (p. 370). More recently, Ndikumana & Boyce estimated that: "During 1970–96, roughly 80 cents on every dollar that flowed into [sub-Saharan Africa] from foreign loans flowed back out as capital flight *in the same year*" [89](p. 122, emphasis added). They also calculate that the accumulated value of flight capital from 25 African countries between 1970 and 1996, plus imputed interest earnings, was considerably *higher *than the entire value of the combined external debt of those 25 countries in 1996 [90]. In other words, taking into account privately held as well as public assets, those African countries should be regarded as net creditors rather than debtors vis-à-vis the rest of the world. Using a similar methodology, Beja [91] estimates the accumulated value of flight capital from Indonesia, Malaysia, the Philippines and Thailand over the period 1970–2000 at $1 trillion, occurring not only during periods of financial crisis but also during periods of economic growth and stability.

A further precondition for the occurrence of debt crises is so basic that it is sometimes overlooked. Banks, national governments and multilateral institutions such as the World Bank have been willing, almost without exception, to accord leaders of developing countries what philosopher Thomas Pogge has called the "borrowing privilege": the right to incur debts on behalf of those they rule without having to defend the legitimacy of their rule. The borrowing privilege is accorded even to leaders who have taken power by force or deceit, maintain it by extreme repression, and are not accountable to citizens in any meaningful way [92].

The impacts of debt crises cannot be understood without considering structural adjustment: a term that entered the international development lexicon in 1980, when the World Bank initiated loans, normally in conjunction with stabilization loans from the IMF, that enabled recipient countries to reorganize their economies in order to increase their ability to repay external creditors. The urgency of such lending grew after 1982, when Mexico's announcement that it was prepared to default on loans made by major US banks raised concern about the stability of financial systems in the industrialized world. The conditionalities attached to World Bank and IMF loans, and to the associated rescheduling of loan payments, emphasized reduction of subsidies for basic items of consumption such as food; rapid removal of barriers to imports and foreign direct investment; reductions in state expenditures, particularly on social programmes such as health, education, water/sanitation and housing; and rapid privatization of state-owned enterprises, on the presumption that private service provision was inherently more efficient, and that proceeds from privatization could be used to ensure debt repayment [93,94]. In other words, the World Bank and IMF promoted multiple, more or less coordinated domestic policies of integrating national economies into the global marketplace. In keeping with widespread usage (see e.g. [94]), structural adjustment here refers to the entire set of domestic policies adopted to reorganize national economies in response to these demands.

Research on health-related impacts of structural adjustment confronts at least three design problems.

First, implementation of conditions attached to World Bank and IMF loans was often incomplete [95] – leaving open at least the theoretical possibility that if the reforms in question had been undertaken even more aggressively, outcomes might have been more favourable. However, the recent history of market-oriented development policy in the two regions of the developing world where it has been pursued most aggressively, Latin America and Africa [96,97], calls this claim into question. So too does the pattern of magnification of inequality through labour market outcomes that has resulted from domestic marketization and export orientation [68,98].

Second, it can be difficult to separate effects of structural adjustment from those of the globalization-related economic crises that preceded and led to engagement with the World Bank and IMF.

Third, and relatedly, every assessment of public policy effects relies implicitly or explicitly on a counterfactual: an alternative state of the world against which the state of the world post-introduction of the policy in question is compared. If structural adjustment is compared with the continuation of business as usual, which would in many cases have involved (continued) hyperinflation and the isolation of countries from international financial markets, then structural adjustment may appear as the least destructive option. On the other hand, if the comparison is with an alternative set of policy options that would have given priority to meeting basic needs, then conclusions about the necessity and desirability of structural adjustment are likely to be less sanguine. For countries highly exposed to the international economy, this counterfactual requires further assumptions about an alternative international order at least partly driven by solidarity or conceptions of obligations that cross national borders – a point to which we return in the third and final article of the series.

A review of studies of the health effects of structural adjustment carried out for the Commission on Macroeconomics and Health [99] found a preponderance of negative effects among 76 studies identified, especially with respect to Africa. This review understated the case against structural adjustment because of incomplete sampling of the literature: the authors' review of the country cases from the "adjustment with a human face" study was cursory, and they did not consider ethnographic studies (e.g. [100-102]) and country-level participatory assessments (e.g. [103]) that shed considerable light on the human consequences of adjustment policies. A larger literature, in much of which a "social democratic" counterfactual [104](p.150) is implicit, describes negative effects of structural adjustment on SDH, but does not extrapolate from the conclusions to generate predictions or hypotheses on health outcomes (for illustrative examples see [94,94,103,105-110]).

A stylized summary is that structural adjustment operated on SDH both directly and indirectly. To illustrate, cuts in food subsidies and in government wages and employment had direct negative effects on access to nutrition and on household income. Import liberalization measures may also have had negative impacts on social structure mediated by labour markets, as livelihoods were lost to low-cost imports. The major effects on social structure, which are often difficult to trace to specific elements of structural adjustment programs (say, to import liberalization as opposed to cuts in state employment) have to do with poverty, income inequality and changing gender relations: for example, the disproportionate impact both on women's incomes and on their household activities (see e.g. [70,110]). Poverty and economic insecurity, in turn, have multiple effects on exposure and vulnerability, mediated by such factors as housing, working conditions, and access to nutrition and education. Structural adjustment also had important equity-related effects on health systems, by way of expenditure reductions and implementation of cost recovery measures.

It is difficult to separate impacts on SDH of domestic policies that were adopted in specific response to lender conditionalities from those adopted in response to the broader diffusion of market-oriented policy ideas. However, the policy changes undertaken as part of structural adjustment programs, which can be generically described as marketization or (re)commodification [63,111], are congruent with the market-oriented policy shifts that are a key element of globalization more generally [78,94]. Ideally, it would be useful to know how much of a country's social and economic policy orientation in year *x *can be attributed to responses to the World Bank and IMF, and how much to national decision-makers' interpretation of the available options within an international economic context over which they may have minimal influence. However, even if it were answerable this question would not alter the fact that if we want to know how globalization affects SDH by way of marketizing domestic social and economic policy, then research on structural adjustment is valuable independent of specific historical connections between lender conditionalities and policy responses. Indeed, given the breadth and depth of structural adjustment conditionalities, it may be the single most important body of evidence available.

That body of evidence is also valuable prospectively. Poverty reduction has replaced structural adjustment in the official vocabulary of the World Bank and the IMF, but similar macroeconomic policy directions can be observed in the Poverty Reduction Strategy Papers (PRSPs) that must be approved by the World Bank and IMF before countries can receive debt relief under the Heavily Indebted Poor Countries (HIPC) initiative and its successor program, the Multilateral Debt Relief Initiative (MDRI), both of which are discussed in the third article in the series. Increasingly, PRSPs are also required before a much larger number of countries can receive grants or concessional loans (i.e., loans at below-market interest) from the World Bank or funding from national development agencies [112,113]. The potential benefits of PRSPs include the explicit identification of poverty reduction as an objective of government policy, requirements for civil society participation, and other administrative conditions. As one specific illustration, Zambia's PRSP requires that District Health Management Boards actually receive at least 80 percent of their specified annual budgets [114](¶20), which apparently had not been the case in the past. On the other hand the macroeconomic policy content of PRSPs may be unduly influenced by lender preferences because of previous country experience with World Bank and IMF conditionalities [115](p. 26–31): in other words, the commitments of even the best-intentioned governments to poverty reduction may, understandably under the circumstances, be tempered by what they think these institutions want to hear [116].

### Cluster 4: Financial liberalization and financial crises

These are examples of overt conditionalities. However, "implicit conditionality" [117] created by the expectations of investors may be at least as important as an influence on public policy. Increased volumes of foreign direct investment (FDI) in production facilities have been accompanied by vastly more rapid growth in portfolio investment: in publicly traded shares, bonds, and an expanding range of financial instruments generically described as derivatives. Whereas the value of global FDI flows was $1.2 trillion for all of 2006 [118], the *daily *value of foreign currency transactions is now estimated at $1.9 trillion [119]. Financial liberalization exposes national economies to the uncertainties created by large and volatile short-term capital flows [120], instantiating Giddens' [121](p. 64) identification of globalization as "an intensification of world-wide social relations which link distant localities in such a way that local happenings are shaped by events occurring many miles away and vice versa." Unlike the imposition of conditionalities by the World Bank and IMF, large-scale disinvestment in response to apprehensions about the viability of a particular national economy or currency requires no formal coordination, still less any kind of conspiracy. It requires only reliance on similar sources of information, such as credit rating agencies [122], and comparable levels of risk aversion on the part of individual private investors and portfolio managers.

The effects of large-scale disinvestment and the resulting financial crises on the 'real economy' and on SDH have been devastating, undermining the livelihoods of hundreds of millions of people as national currencies lost 50 percent or more of their value relative to hard currencies like the US dollar; purchasing power evaporated; and restoring the country's creditworthiness in the eyes of investors with the option of placing their assets elsewhere took priority over meeting basic needs domestically. This happened in Mexico in 1994–95, as Mexican and foreign investors shifted their assets out of Mexican government debt securities and forced further devaluation of the peso [123]; in south Asia in 1997–98, even among the so-called Tiger economies that were counted among globalization's success stories, after flight from the region's currencies began with speculation against the Thai baht [124-126]; and most recently in Argentina in 2001–02 [127]. In an especially striking instance of long-distance effects, investor concern about the stability of all developing country currencies in the wake of crises in Korea (late 1997) and Russia (early 1998) led to a selloff of Brazilian assets that forced a currency devaluation. This happened even though connections between Brazil's economy, and the economic lives of most Brazilians, with events in Korea or Russia were minimal [128,129]. Predictably given existing national and household-level distributions of power and access to resources, the impact of financial crises is often felt first, and worst, by women [130,131] – suggesting, as do many other aspects of globalization's impacts, the need for a gender-specific approach to macroeconomic and social policy responses on the part of researchers, national governments, and multilateral institutions alike [64,65,132,133].

The effects of financial crises may sometimes be magnified by contractionary economic policies, financial sector liberalization or labour market 'reforms' undertaken in response, either as the price of bailouts from the IMF and industrialized country governments or as an attempt to restore credibility with investors who have shifted their assets elsewhere [134-138]. If financial liberalization promotes growth [138], it may be at the cost of an increase in economic inequality [136]; conversely, in at least one case (that of South Korea) financial crises actually generated political support for a limited expansion of the welfare state [139]. Less amenable to conflicting interpretations are the findings of a comparison of financial crises in 10 countries [140] that showed that employment recovers much more slowly than GDP in the aftermath of financial crises, exacerbating their effects on social stratification and the vulnerabilities associated with economic inequality and insecurity. A further effect is that the value of external debt obligations denominated in dollars or other hard currency climbs with any devaluation, creating additional economic constraints on domestic public sector budgets [141] in such areas as health care and education.

Using a schematic analogous to one developed with respect to globalization and HIV infection, described in the first article of this series [142], Hopkins [143] cites research showing that reductions in household income as a result of financial crises in Indonesia, Thailand and Malaysia during the late 1990s led to reduced food intake, health care utilization and education expenditure. Indicative of the potential health effects is a Korean national survey that found substantial increases in morbidity, and decreases in health service utilization, following the 1997 currency crisis [144]. Simultaneously, declining tax revenues led to lower public expenditure on health and education. The combined effect was to increase mortality and reduce longevity – a disturbing reprise of the findings of UNICEF's original *Adjustment With a Human Face *study [145]. Although the depth and duration of financial crises and their impacts on SDH vary considerably, asymmetry characterizes their origins and impacts both domestically (in terms of economic effects and the distribution of opportunities to escape them) and internationally (in terms of the global shift in power toward the owners of internationally mobile financial assets).

### Cluster 5: Cities restructured by the global marketplace

Long-distance effects of quite a different kind are evident in changing patterns of urban form and settlement, and assume special importance given the estimate that the world's urban population will have grown by more than two billion people between 2005 and 2030. Almost all of this growth will occur in countries with limited resources to provide urban and peri-urban infrastructure that is taken for granted in most of the industrialized world [146]. A consistent pattern in the transformation of cities and metropolitan areas by transnational economic integration, in countries rich and poor alike, is that gaps between economic winners and losers grow, based on their position within the global economy and the basis of their connection (or lack of connection) to it. Statistics on income disparities capture only part of the picture. Castells' description of the urban impacts of globalization in terms of a "space of flows" [147] is valuable because it reminds us that 'connectedness' to the networks of investment and information that characterize the globalized economy may have nothing to do with proximity as viewed on a road map. Castells observes that urban districts whose residents are not part of the "process that connects advanced services, producer centers, and markets in a global network" can become "irrelevant or even dysfunctional: for example, Mexico City's *colonias populares *(originally squatter settlements) that account for about two thirds of the megapolitan population, without playing any distinctive role in the functioning of Mexico City as an international business centre" [147](p. 380–381). Thus, large metropolitan areas will contain substantial "local populations that are either functionally unnecessary or socially disruptive" [147](p. 404).

Spatial divisions that reflect or reinforce the pattern of gains and losses from globalization arise in a variety of ways. In parts of the industrialized world, they have been initiated by large-scale job and income losses and economic polarization associated with deindustrialization [148-151]. Even in the immensely wealthy United States, some cities with economies built on manufacturing lost half to three-quarters of their manufacturing jobs in the second half of the 20^th ^Century [152-154], with devastating effects on economic opportunities and the social fabric [155,156]. Urban 'revitalization' may include not only policies that favour more desirable (read: higher-income) residents, but also reconfiguration of urban space in pursuit of profitable commercial development and tourism revenues, similarly leading to displacement of residents and sometimes the literal enclosure of public spaces (see e.g. [157-162]). Residential segregation deepens through gentrification, suburbanization, and the creation of fortified enclaves with separate private systems of service provision, while those less able to pay are shifted to less desirable locations and rely on inferior services. Policy choices with special significance for the boundaries of inclusion/exclusion involve transportation, specifically the balance between public transit and car-centred development (see e.g. [160,163,164]). In this and other cases, access to essential resources is often determined by individual households' ability to pay or by group/neighbourhood attractiveness as a market. Poverty may be criminalized [165,166]. These processes are documented in an indispensable UN Habitat synthesis on *Cities in a Globalizing World *[167], hence the lack of more extensive references here.

Bidding contests for urban spaces, which epitomize the interplay of global power relations and local opportunities, are paralleled by contests over locationally valuable non-urban resources, notably those associated with the expanding business of tourism. These contests can exclude current, low-value or low-productivity users of a resource either by degradation, e.g. by using surface or ground water as a sink for the disposal of toxic wastes [168], or by enclosure, e.g. by pricing the use of specific locations and resources out of reach of all but the wealthy [169-171]. The common analytical denominators in these conflicts are: (a) in the global marketplace, some resources simply command too high a price to be used for the basic needs of people with limited purchasing power, and (b) domestically, the polarization of income and wealth that accompanies economic integration shifts the political allegiances of decisive political pluralities in the direction of private service provision rather than collective action. Asymmetry, once again.

### Cluster 6: Globalization, natural resources and environmental exposures

The global marketplace for natural resources and services or amenities provided by the natural environment creates an important and complex set of influences on SDH. Stonich and Bailey [172](p. 23–24) argue that pressure to increase export earnings leads governments to promote "export-oriented aquacultural development regardless of the social and environmental consequences," creating situations in which "the increasing use of low-value fish species in the production of fishmeal for aquacultural feeds in effect puts the poor in competition with shrimp," and with the rich consumers who can afford to buy them (see also [173]). This is one instance of a pattern noted by the health synthesis of findings from the Millennium Ecosystem Assessment (MEA) project, which explicitly recognized economic globalization as one of the drivers of change in ecosystems and human well-being by way of various causal pathways: "Historically, poor people disproportionately have lost access to ecosystem services as demand from wealthier populations has grown" [174](p. 28). The ecosystem services in question may themselves be essential to health, or else may be essential sources of livelihood the loss of which leads to economic insecurity and deprivation. The difference globalization makes is that winning bidders may be half a world away, as in Stonich's aquaculture example and in the case of markets for tropical timber, oil in Nigeria (where abundant resource revenues have failed to improve the grinding poverty and poor health status of much of the country's population), and coltan and other minerals in the Democratic Republic of the Congo [175-181].

As globalization increases aggregate demand for marketable resources and ecological services, it simultaneously fosters policies and institutions that facilitate control over gains and losses across entire regional economies by local elites and the dominant actors in global commodity chains (see e.g. [177,178]). Analysis of investment in developing countries by transnational logging companies in response to increasing global demand for tropical timber was strongly critical of the sustainability of forest management practices, and further noted that: "Where analysis is available... the economic benefit is minor, even in the short-term, and certainly far less than it could be if contracts were structured and negotiated differently. While large amounts of capital are involved, the revenue to national treasuries can be small because most of the profits leave the country or accrue in the hands of very few, often already wealthy and powerful local people" [179](p. 29, citations omitted). Transnational mineral firms are often the beneficiaries of large-scale financial support from export credit and insurance agencies in their home countries [182,183] – an element of global influence that appears to have received little research attention outside a rather specialized community of CSOs. In such cases, global asymmetries of economic power are reflected in extreme inequalities in the distribution of benefits domestically.

The MEA health synthesis noted another set of differential exposures and vulnerabilities: "Poor populations are more vulnerable to adverse health effects from both local and global environmental changes" [174](p. 27), first of all because they are more likely to be exposed to hazards from which the rich can remove themselves. Disasters in Bhopal and New Orleans provide dramatic evidence of this point, as do the routine conditions of urban life for literally hundreds of millions of people worldwide [184]. It is estimated that more than 850 million people now live in slums, with the number projected to rise to 1.4 billion in 2020 in the absence of effective policy interventions [146]. Slum residence is an imperfect, but nevertheless useful proxy for exposure to urban environmental hazards including infectious disease related to inadequate sanitation and industrial pollution, as well as other quotidian risks exemplified by the collapse of a rain-soaked open rubbish dump that killed some of the residents of Manila's informal settlements in 2000 [185,186].

Some studies find a clear pattern of migration of hazardous industries to lower-income countries, notably to export processing zones (EPZs) [50,52]. Other, quantitative studies that do not focus on particular regions suggest that evidence for the emergence of industrial "pollution havens" is equivocal or absent [187,188]. An impressionistic assessment of such 'negative' findings is that many are compromised by (a) failure to focus on the global restructuring of production within specific industries or sectors; (b) concentration on foreign direct investment (FDI), without considering contractual arrangements such as outsourcing that are not recorded in FDI statistics but are extensively described in the literature on commodity or value chains; (c) inability to distinguish causal effects of lax environmental regulation on relocation of production (what the pollution haven hypothesis is all about) from those of other variables, such as low wages and flexible working conditions, that tend to operate in parallel; and (d) failure to distinguish between changes in pollution exposures attributable to industrial processes and to such factors as increased vehicle traffic. (Pollution exposures resulting from increased vehicle traffic may also be consequences of globalization, e.g. as it supports a growing 'middle class' and associated settlement patterns, but these consequences are analytically separable from the industrial migration or pollution havens hypotheses.) Substantial evidence also exists of the emergence of a global trade in hazardous wastes, with disposal in low-income countries becoming increasingly attractive and met with policy responses that are at best only partially effective [189-192].

In the background is the question of whether such environmental changes and their health impacts should be regarded as normal, in the sense that they are comparable to those undergone by the industrialized countries at comparable stages of their own economic development. Evidence of the extent to which contemporary technology allows for "technological leapfrogging" [193] and "dematerialization" [194], which avoid many environmentally destructive forms of industrial production and consumption, suggests that this conclusion should be rejected. However, environmental and resource impacts can alternatively be considered with reference to a green counterfactual that assumes transfer of clean technologies on favourable terms, along with serious efforts by the industrialized economies to reduce their consumption of natural resources and ecological services, and to adopt policies that minimize negative environmental and resource impacts outside their borders. Thus, globalization's negative effects on SDH that operate by way of the environment, like those that operate in other ways, must be regarded primarily as consequences of political choices and avoidable failures of governance.

### Cluster 7: Marketization of health systems

Health care interventions that would be taken for granted in the industrialized world are routinely unavailable, or available only to rich minorities, outside it [100,195,196]. As a result, literally millions of preventable deaths occur every year. Multilateral institutions like the World Bank have historically worsened this situation by promoting and reinforcing a market-oriented concept of health sector reform (HSR) that strongly favours private provision and financing [66,197,198]. Reductions in public sector health spending, introduction of user fees, and other cost recovery measures aimed at making health systems financially self-sustaining were often mandated as part of structural adjustment conditionalities [101,199-202] despite their regressive impacts. (Although the World Bank has now acknowledged the inequity of relying on user fees and private purchase of health care in its commendably equity-oriented 2006 *World Development Report *[203](p. 146–149), it continues to promote private health insurance in developing countries in conjunction with the financial services industry [204].)

Official user charges in some instances may replace informal, and even more inequitable patterns of side payments demanded by care providers or suppliers of medicines [205-207], but their effectiveness in generating revenue is limited, while access to health care for the poor and otherwise vulnerable often deteriorates (for reviews see [208-212]; key case studies include [201,213-223]). This deterioration occurs because very large numbers of people simply cannot afford necessary health care [224-227]. Ethnographic research and the experiences of front-line care providers [100,195,228] support the conclusion that the issue is often not one of unwillingness to pay, but rather of inability to pay, and understandable reluctance to sell off assets that may be critical to the household's economic survival [224,229].

Marketization of health systems may also compromise progress in other health-related areas such as poverty reduction, as medical costs and lost earnings associated with serious illness create "medical poverty traps" [225]; as noted earlier, these effects will not be reflected in national poverty statistics when poverty is defined with reference to household consumption [14]. Viet Nam is often cited as an example of the poverty-reduction benefits of embracing the global marketplace, yet health indicators reflect the widespread 'double burden of disease' phenomenon in which infectious diseases largely eradicated in the industrialized world coexist with rapidly rising incidence of chronic non-infectious diseases and injuries from such causes as road traffic accidents [230]; opening up of domestic markets has been accompanied by the dismantling of relatively equitable systems for social and economic provision [215,220,230]. In a much larger country, whatever the economic gains from China's domestic social and economic policy reforms, a survey of several Chinese provinces found that the percentage of women with insurance coverage for prenatal and delivery services fell from 58.3 percent in 1989 to 34.7 percent in 1997; overall access to insurance coverage, already available to just one in four Chinese in 1989, continued to decline slowly through the 1990s [231]. The public share of health expenditures fell by over half between 1980 and 1998, almost trebling the portion paid by households [219], leading to the growth of private delivery systems for those who could afford them, and increased cost-recovery for services that were still under some form of public health insurance. The result was an increase in the number of people who fell into poverty by exhausting their income and savings to pay for medical treatment [219] and a slowdown in China's population health improvements, particularly infant mortality and life expectancy [231]. It remains to be seen whether recent initiatives to reverse deterioration in access to health care for the poor, notably in rural areas, will be effective [232](p.15–19), [233].

Four further dimensions of globalization's effects on health systems must be considered.

First, despite a WTO interpretation of TRIPS that limits patent protection for essential medicines, concern remains about the effectiveness of this interpretation as reflected both in national legislation (in countries with substantial pharmaceutical industries) and trade policy practice (in countries without) [234].

Second, commitments made under the General Agreement on Trade in Services (GATS) and bilateral and regional agreements such as NAFTA have the potential to lock in privatization initiatives against future governments' efforts to expand public provision or insurance [235,236], although disagreement exists about the seriousness of this prospect.

Third, the 'brain drain' of health professionals from developing countries, in particular those in sub-Saharan Africa, to industrialized countries where they can earn far more is now recognized as one of the most serious problems confronting health systems [237-239]. Solutions remain elusive because the situation reflects a bidding contest for the services of health professionals that is analogous in many respects to the bidding contests for urban space and locationally valuable resources described in the preceding section.

Fourth, health research priorities based on the availability of private funding are highly problematic on health equity grounds. Private for-profit firms (mainly pharmaceutical firms) now outspend governments worldwide on health research [240]; the Bill and Melinda Gates Foundation had more money at its disposal than the World Health Organization [241] even before its recent windfall from the assets of Warren Buffett; and public funding agencies in many industrialized countries link their priorities to the anticipation of commercial returns. The result is a major mismatch between health research priorities and the main contributors to the burden of disease outside the industrialized world. Of 1556 new drugs (new chemical entities) marketed between 1975 and 2004, only 21 were for neglected diseases, malaria and tuberculosis [242]. This figure does not reflect a recent increase in research activity related to 'neglected diseases' that has not yet led to new marketable products [243]; neither does it take into account the potential applicability to developing country contexts of much research that addresses chronic non-communicable diseases once largely confined to the industrialized world, as the double burden of disease phenomenon becomes more significant.

### Coda

The story of globalization's impacts on health that is outlined here is not a cheerful one. Critics might argue that we have failed to consider the parallel increases in wealth and health worldwide during the second half of the last century, and their historical point is undeniably accurate. Economic growth has indeed made a large number of concrete contributions to SDH, for example by supporting the transition to cleaner fuels for cooking and a resulting decrease in pollution-related respiratory diseases, even as this transition remains elusive for large numbers of the world's people [244]. Further, as noted in the first article of the series, a leading researcher on growth and health warns that "economic growth, by itself, will not be enough to improve population health, at least in any acceptable time." He continued with the observation that: "As far as health is concerned, the market, by itself, is not a substitute for collective action" [245]; for elaboration see [246]. Even leaving aside this observation, the past is not always a reliable guide to the future. Grand narratives about globalization and health that rely on one or two measures of either global market integration or health yield unconvincing conclusions, and their failure explicitly to address the multiple asymmetries that characterize contemporary globalization means they provide little guidance for policy. In this article we have chosen to focus our gaze more precisely. Our findings are not definitive, but they point to a certain urgency in rearranging the rules under which contemporary globalization is unfolding. A preliminary description of key elements of the necessary rearrangement is provided in the third and final article of this series.

## Competing interests

The author(s) declare that they have no competing interests.

## Authors' contributions

The authors contributed equally to the conception and design of the study; acquisition, analysis and interpretation of data; and drafting of the manuscript. Both authors have read and approved the final manuscript.
